# Advanced Hybrid Strategies of GelMA Composite Hydrogels in Bone Defect Repair

**DOI:** 10.3390/polym16213039

**Published:** 2024-10-29

**Authors:** Han Yu, Xi Luo, Yanling Li, Lei Shao, Fang Yang, Qian Pang, Yabin Zhu, Ruixia Hou

**Affiliations:** 1 Department of Cell Biology and Regenerative Medicine, Health Science Center, Ningbo University, Ningbo 315211, China; 15981559175@163.com (H.Y.); 18725486331@163.com (X.L.); 15880739343@163.com (Y.L.); scholar.vincenty@outlook.com (F.Y.); zhuyabin@nbu.edu.cn (Y.Z.); 2 Research Institute for Medical and Biological Engineering, Ningbo University, Ningbo 315211, China; shaolei1@nbu.edu.cn

**Keywords:** GelMA, composite hydrogels, bone tissue engineering, bone regeneration

## Abstract

To date, severe bone defects remain a significant challenge to the quality of life. All clinically used bone grafts have their limitations. Bone tissue engineering offers the promise of novel bone graft substitutes. Various biomaterial scaffolds are fabricated by mimicking the natural bone structure, mechanical properties, and biological properties. Among them, gelatin methacryloyl (GelMA), as a modified natural biomaterial, possesses a controllable chemical network, high cellular stability and viability, good biocompatibility and degradability, and holds the prospect of a wide range of applications. However, because they are hindered by their mechanical properties, degradation rate, and lack of osteogenic activity, GelMA hydrogels need to be combined with other materials to improve the properties of the composites and endow them with the ability for osteogenesis, vascularization, and neurogenesis. In this paper, we systematically review and summarize the research progress of GelMA composite hydrogel scaffolds in the field of bone defect repair, and discuss ways to improve the properties, which will provide ideas for the design and application of bionic bone substitutes.

## 1. Introduction

Bones serve vital functions in the body, including providing support, protection, facilitating movement, and acting as a storage reservoir [1]. However, their ability to self-heal is limited. Bone defects, which arise from trauma, tumors, inflammation, necrosis, or surgical interventions, are influenced by numerous systemic and local factors. Currently, 5% to 10% of bone defects remain unhealed over extended periods, a concern that is exacerbated by the global aging population [2]. This persistent issue not only strains healthcare resources but also causes significant patient suffering. Globally, approximately 37 million fragility fractures occur annually in individuals over the age of 55—equating to 70 fractures every minute—resulting in a substantial economic burden [3]. While small bone defects may heal naturally, large defects (greater than 2 cm or involving over 50% of the bone’s diameter) typically require surgical intervention. Currently, the gold standard for the surgical treatment of bone defects remains autologous bone grafting, which avoids immune rejection and adapts quickly to the organism [4]. However, the lack of bone donors and the high cost severely hampers its clinical applications. In addition, fillers for repairing bone defects include allografts, xenografts, and bone graft substitutes. Among them, bone graft substitutes have many advantages, including ease of use, fewer adverse effects and lower prices, etc. [5,6]. Commonly used bone graft substitutes today include metals and bioceramics. However, the ideal biomaterial is one that mimics the structure of bone and the environment of the natural extracellular matrix (ECM), is biologically active, and has a certain mechanical strength. Seeking suitable substitutes for bone grafts is a challenge in the treatment of bone defects.

Developments in bone tissue engineering have provided more alternatives to bone grafting. Bone tissue engineering uses engineered scaffolds combined with growth factors, and integrated cells to develop bone graft substitutes, using chemical or physical methods to modulate the cell growth microenvironment and promote bone tissue regeneration. Developments in bone tissue engineering have provided more alternatives to bone grafting. In particular, hydrogels, as hydrophilic polymer scaffolds, can provide a three-dimensional polymer network similar to that of natural bone, which is suitable for cell growth and the transfer of biologically active molecules, making them a potential choice for bone repair and bone regeneration [7,8].

Hydrogels are polymers capable of swelling in water and possessing elasticity close to that of natural tissues, with good biocompatibility and degradability [9]. During the process of bone tissue neogenesis, the ECM plays an important role in signal transduction and nutrition exchange between the budding and primitive tissues [10]. Hydrogels are able to mimic the complex properties of ECM, and the construction of hydrogel scaffolds for tissues, such as gastrointestinal, skin, and bone, has been reported for these scaffolds, which demonstrate remarkable abilities to support cell growth, proliferation, and differentiation, and to promote tissue regeneration and healing [11,12,13,14]. Moreover, because of their structure, hydrogels are better able to load and release growth factors and drugs. The materials, crosslinking methods, and hybridization can be varied to obtain hydrogels that meet specific needs [15].

Natural biomaterials are promising for the synthesis of hydrogel scaffolds, such as collagen, fibronectin, alginate, chitosan, etc., which are widely used in bone regeneration. In contrast, gelatin, as a denaturation product of collagen, has attracted much attention due to its possession of arginine–glycyl–aspartate (RGD) and matrix metalloproteinase (MMP) sequences, the former being able to promote cell–skeleton interactions and the latter being able to degrade collagen. GelMA is a methacrylic anhydride-modified gelatin derivative with a controlled chemical network, high cellular stability and viability, good biocompatibility and degradability, and contains properties similar to natural ECM, allowing cell adhesion and remodeling, which is widely used for tissue engineering applications [16,17].

**Scheme 1:**
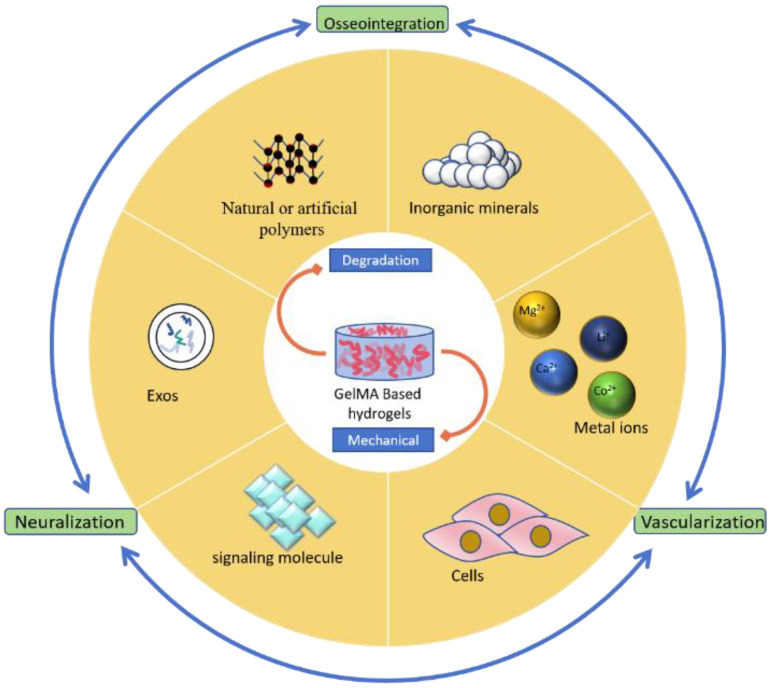
Schematic description of GelMA hydrogels hybridized with different materials, improve their shortcomings and endowing them with the functions of osseointegration, vascularization, and neuralization [18].

In this paper, we introduce GelMA hydrogels from a functional perspective and analyze the effects of the incorporating different materials on the properties of GelMA hydrogels. Combined with existing studies, we explore the application of GelMA for bone defect repair, providing different hybridization strategies as well as the prospects and challenges of GelMA in this field, as shown in Scheme 1, thus providing ideas for the design of GelMA composite biomaterials.

## 2. GelMA Hydrogels

Gelatin, a hydrolyzed collagen product with bioactive motifs, is a key material in tissue engineering and regenerative medicine. While it naturally has a low melting point and dissolves at body temperature, it can be chemically cross-linked to stabilize and connect protein chains. Van Den Bulcke et al. enhanced gelatin by introducing methacryloyl groups, preserving its RGD and MMP sequences and adding photocrosslinkable properties, improving its use in tissue engineering.

GelMA, produced from porcine or bovine gelatin and methacrylic anhydride (MA) in phosphate buffer solution (PBS) at 50 °C, shows varying degrees of substitution (DS) depending on the MA content and pH [19]. Eva Hoch et al. demonstrated that using different MA to gelatin ratios (1:1.5, 1:2, 1:10, or 1:20) at a pH of 7–7.4 resulted in DS values from 67.2% to 98.2% [20]. Shirahama et al. further found that a pH of 9 led to higher DS compared to other pH levels [21].

**Figure 1:**
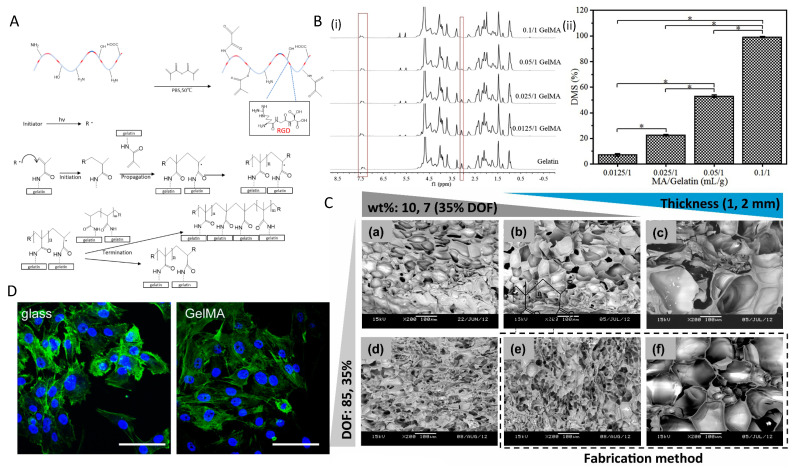
Synthesis of gelatin methacryloyl (GelMA) hydrogel and its properties. (**A**) Scheme for the preparation of photocrosslinked GelMA hydrogel [18]. Copyright © 2015 Elsevier; (**B**) (**i**) 1 HNMR spectra of unmodified gelatin and GelMA with different DMS (dimethyl sulfide) values. The left and right red rectangles represent the signals of aromatic amino acid and lysine amino acid, respectively; (**ii**) the relationship between DMS of GelMA and the MA–gelatin feed ratio (* *p* < 0.05) [20]. Copyright © 2015 Springer Nature; (**C**) scanning electron microscopy (SEM) images of homologous series of GelMA hydrogels showing the influence of hydrogel %DOF, monomer concentration, and sample geometry: (**a** vs. **b**) 10 vs. 7 wt%, 35 % DOF, 1mm thick. (**b** vs. **c**) 1 vs. 2 mm thick, 10 wt%, 35 % DOF. (**a** vs. **d**) 35 vs. 85 % DOF, 10 wt%, 1 mm thick. (**d** vs. **e**) 10 vs. 7 wt%, 85 % DOF, 1 mm thick. (**e** vs. **f**) 2 mm thick photopolymerized in Teflon mold vs. between glass slides. Scale bars: 100 μm [24]. Copyright © 2013 Wiley; (**D**) attachment of MG63 osteoblasts on glass and GelMA [18,25], copyright © 2017 IOP Publishing.

The synthesized GelMA is dialyzed in pure water to remove low molecular weight impurities and then freeze-dried for storage. GelMA can be rapidly cross-linked by UV or visible light irradiation in the presence of photoinitiators to form a stable three-dimensional polymerized network [22,23] (Figure 1). The hydrophilic three-dimensional network allows for the exchange of water, nutrients, and metabolic wastes between the defect site and normal cells, while providing enough space for tissue growth as the hydrogel matrix degrades [24].

GelMA is widely used in many fields, such as nerve repair, cartilage regeneration, and organoid construction [26,27,28]. In research on the treatment of bone defects, it works in various forms, such as composite scaffolds, drug carriers, cellular carriers, and bionic osteochondral membranes [29]. McBeth et al. demonstrated that GelMA hydrogel with porous features was able to trigger the differentiation of human osteoblasts in the absence of an exogenous bone differentiation medium [25]. However, GelMA hydrogel alone does not provide the mechanical strength, osteogenic factors, and mineral ions, such as calcium and phosphorus, required for bone reconstruction. It also has the disadvantages of a long gelation time, short degradation time, and high swelling rate when used as a guided bone regeneration (GBR) material, so it needs to be combined with other materials to obtain specific functionality [30]. For example, the micromachining of GelMA hydrogels allows control of their 3D microstructure, creating unique patterns and morphologies, and provides an ideal platform for controlling cell behavior, studying cell–biomaterial interactions, and designing tissues [18].

## 3. Enhanced Mechanical Performance

The compression modulus ranges of GelMA hydrogel and bone are 1.82 kPa–4.83 kPa [31] and 2.0 GPa–18 GPa [32], respectively. Because the compressive modulus of bone is much higher than that of GelMA hydrogel, their mechanical properties do not match. Therefore, GelMA alone cannot be directly applied as a bone scaffold, so in order to match the mechanical properties with bone tissue, researchers have explored many methods, such as changing the fabrication process of GelMA, hybridizing it with nanomaterials, and enhancing its intermolecular dynamics [33].

### 3.1. Changing Experimental Parameters

**Figure 2:**
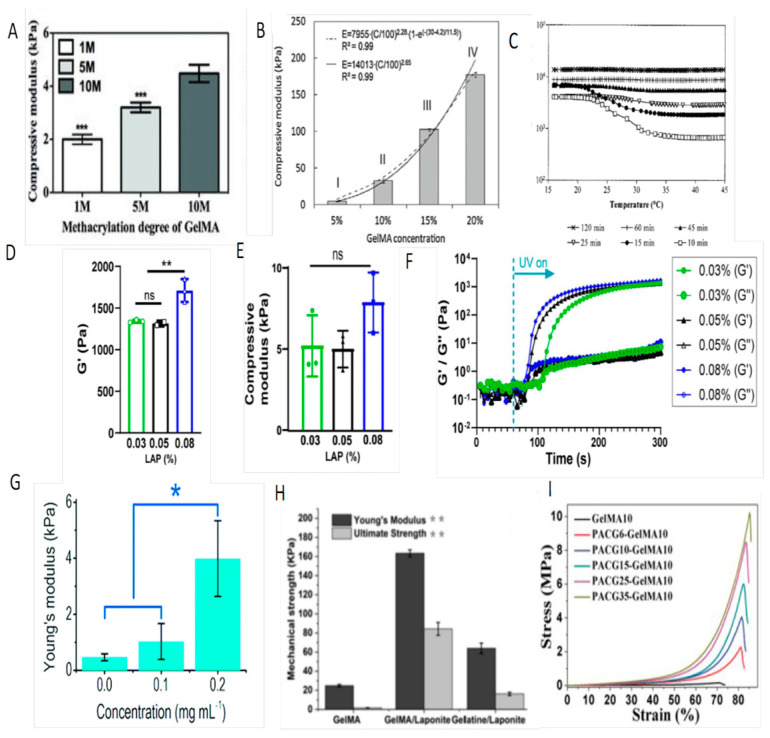
Enhanced mechanical performance. (**A**) Compressive modulus of hydrogels made of GelMA with various DM [34]. Copyright © 2012 Wiley; (**B**) compressive modulus of hydrogels made of different concentrations of GelMA [35]. Copyright © 2013 Wiley; (**C**) effect of UV exposure time (10–120 min) on the temperature dependence of the elastic modulus [23], copyright © 2000 American Chemical Society; (**D**) effect of LAP concentration on G’-plateau; (**E**) effect of the laponite in different ink on the mechanical strength; (**F**) time sweep of storage and loss moduli (G’/G’’) [37]; (**G**) Young’s modulus of hybrid hydrogels of 5 wt% GelMA and different concentrations of submicron line [38]. Copyright © 2021 Elsevier: (**H**) Mechanical characterization of acellular printed gel; (**I**) Effect on the hydrogels with 10 wt% GelMA and varied initial concentrations of PACG [38]. Copyright © 2021 IOP Publishing; (ns ≙ *p* > 0.05, * *p* < 0.05, ** *p* < 0.01, *** *p* < 0.001).

Changes in methacrylic acid substitution, GelMA proportion, photoinitiator concentration, and initiation time cause differences in the physical properties of GelMA hydrogels [18]. Ying-Chieh et al. synthesized GelMA hydrogels with substitution degrees of 49.8%, 63.8%, and 73.2%. The mechanical strengths of these hydrogels were 2.0 ± 0.18 kPa, 3.2 ± 0.18 kPa, and 4.5 ± 0.33 kPa, respectively. They found that the compression modulus of GelMA increased with the degree of methacrylation (Figure 2A) [34]. Schuurman et al. used 5%, 10%, 15%, and 20% solutions of GelMA, to make hydrogels under UV light, with an intensity of 2.7 mW/cm^2^ to obtain samples of varying hardness (9.9–183.1 kPa) (Figure 2B). As the concentration of GelMA increased, the hardness of the hydrogels continued to increase [35]. Van Den Bulcke et al. explored how initiator concentration, UV irradiation time, and storage conditions affect the mechanical properties of GelMA hydrogels. They found that within the 0.002% to 0.005% *w*/*v* range, increasing the Irgacure 2959 concentration enhanced the hydrogel’s mechanical strength, but concentrations exceeding 0.025% *w*/*v* resulted in a hard and brittle structure. They also demonstrated that mechanical strength increased with higher UV light doses (Figure 2C) [23].

In a related study, Sergei Butenko et al. fabricated hydrogels using a 20% *w*/*v* GelMA solution, polymerizing it with 0.1% *w*/*v* Irgacure 2959 under 365 nm UV light for 1 min (lo-GelMA) and 5 min (hi-GelMA), resulting in hydrogels with stiffnesses of 3 kPa and 150 kPa, respectively, for treating skin defects in mice. Their study revealed that hi-GelMA significantly increased macrophage M1 activity (24.5% in hi-GelMA vs. 18.1% in lo-GelMA) and M4 activity (13.3% vs. 6.8%), alongside an enhanced neutrophil-driven inflammatory response [36]. The balance between irradiation time and photoinitiator dose is crucial, as is the storage temperature of the material. Notably, storing the hydrogels at 4 °C for 24 h before UV irradiation significantly improved the elastic modulus compared to shorter storage durations [23].

Gehlen et al. investigated how varying concentrations of LAP, from 0.08% to 0.03% (*w*/*v*), influence gel crosslinking and stiffness. At a LAP concentration of 0.8%, the storage modulus is 1.23 times higher, and the compression modulus is 1.56 times higher than those of the other groups. The experiment showed that higher LAP concentrations could accelerate the photocrosslinking and strengthen the stiffness of hydrogels [37].

Consequently, the mechanical strength of GelMA hydrogels can be enhanced by increasing the degree of methacrylic acid substitution, improving the concentration of prepolymers or photosensitizer, and prolonging the light-curing time.

### 3.2. Nanomaterial Hybridization

The incorporation of nanomaterials into the hydrogel network as a dispersed phase within the hydrogel crosslinked polymer matrix can provide rigid support and bridging for the formation of the hydrogel network [28,39]. The conjugation between polymers and nanomaterials allows the mechanical forces exerted by the hydrogel to be transmitted and dispersed through the network, thereby increasing the mechanical strength of the material.

Sadeghian et al. fabricated a metallic glass submicron wire with adjustable composition and physical properties that was structurally stable and doped it with 5 wt% GelMA to produce a hydrogel sample, which significantly enhanced the mechanical strength of the GelMA hydrogels. The stiffness of the hybrid hydrogel doped with metallic glass submicron wires was 1.03 ± 0.64 kPa, significantly higher than that of GelMA hydrogel alone, at 0.47 ± 0.12 kPa, and the mechanical strength is increased by 219%. The metallic glass submicron wires inside the hybrid hydrogel formed a mesh structure, which supported the GelMA to form a more robust 3D structure [39].

Laponite is an inorganic material with a unique disc structure (negatively charged on the surface and positively charged at the edges). Due to its strong potential for inducing angiogenesis and osteogenesis, it is widely used in bone tissue engineering [40,41]. Dong et al. developed a nanocomposite hydrogel consisting of 8% (*w*/*v*) laponite and 15% (*w*/*v*) gelatin methacrylate (GelMA). The results of the study showed that the addition of laponite increased the tensile modulus of the hybrid hydrogel by 9 times and the compressive modulus by 41 times compared to the GelMA hydrogel (Figure 2G–I) [38]. This study further demonstrated that the hybridization of GelMA with nanomaterials enhanced the ultimate tensile strength and ultimate tensile modulus of hydrogels.

### 3.3. Enhancement of Intermolecular Dynamics

The intermolecular forces in the chemical network of GelMA hydrogels are weak, and combination bonds are introduced to enhance the rigidity of the GelMA hydrogel network for better application. The mechanical properties of GelMA hydrogels can be improved by enhancing hydrogen bonding. Gao et al. used 3D printing to synthesize PACG-GelMA hydrogels with GelMA and N-acryloyl 2-glycine (ACG) monomers as bioinks. The multiple hydrogen bonding enhancement of PACG enables the hybrid hydrogel to exhibit excellent tensile strength (up to 0.1 MPa and 1.1 MPa), large stretchability (up to 139% and 245%), high Young’s modulus (up to 143 kPa and 281 kPa), high compressive strength (up to 4.1 MPa and 12.4 MPa), and high compressive modulus (up to 421 kPa and 651 kPa). Additionally, it overcomes the rapid degradation of PACG due to carboxylate group dissociation [42].

Inspired by mussels, polydopamine (PDA) is able to introduce a variety of non-covalent interactions into the hydrogel network to enhance the stretchability and toughness of hydrogels [43]. Gan et al. inserted a dopamine oligomer of methacrylamide (ODMA) into GelMA hydrogels to reduce the strength of the physical chain linkages within the GelMA hydrogel. The ODMA acts as a crosslinking point to connect with the GelMA and to provide dissipated energy during the deformation of the hydrogel, resulting in increased toughness and elasticity. Scanning electron microscopy results showed that the introduction of ODMA into the hydrogel network caused it to exhibit a looser structure, and the pore size of the hydrogel increased with the addition of ODMA. The ODMA–GelMA hydrogel is superior to pure GelMA gel in all mechanical properties: the compressive strength is 5 times higher, and the maximum elongation is 2 times higher. Despite the fact that physical bonds, such as hydrogen bonding and π–π stacking, are sacrificed in this method, the hydrogels show more excellent physical properties [44].

Liu et al. mixed small molecules of 3,4-dihydroxybenzaldehyde into the chemical network of GelMA/phenylboronic acid-modified gelatin hydrogel, in order to introduce boronic acid ester bonds and Schiff base bonds, which acted as a fast “dynamic bridge”, and prepared a high-strength composite hydrogel. Compression test results showed that when the content of phenylboronic acid-modified gelatin reached 15%, the compressive strength reached 2.6 MPa, which is much higher than that of simple gelatin. Meanwhile, the tensile property was also improved [45].

In summary, enhanced intermolecular interactions can lead to excellent mechanical properties in hydrogels.

## 4. Regulating Material Degradation

GelMA hydrogels can be easily hydrolyzed by collagenase I and collagenase II due to gelatin’s RGD and MMP sequences. The regeneration time required for bone tissues with different sizes of defects varies, but all are longer than the degradation time of GelMA. Therefore, hybridized GelMA hydrogel is required to degrade slowly in living organisms to provide mechanical support for the repair of bone tissues, promote the proliferation of bone cells, and facilitate the exchange of materials and information at the site of defects.

Current studies have slowed the degradation of hydrogels by increasing the prepolymer concentration of GelMA, changing the type of photoinitiator, and introducing synthetic organic materials to enhance the degree of bonding. Table 1 summarizes the degradation rates of various GelMA-based hydrogel scaffolds in different solvents, showing how material composition and the presence of enzymes influence the remaining mass over time.

### 4.1. Increasing the Concentration of GelMA Prepolymers

**Figure 3:**
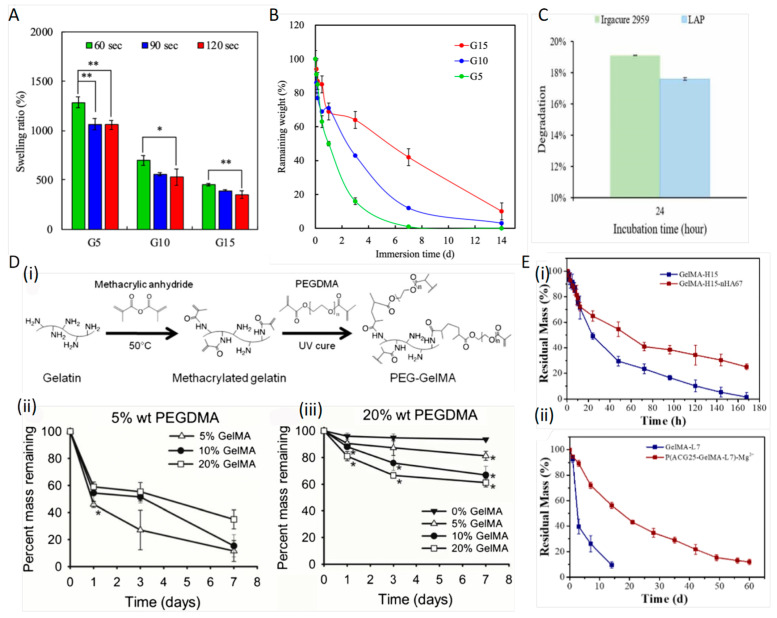
Mitigating the degradation of GelMA-based hydrogels. (**A**) Equilibrium swelling properties of the GelMA hydrogels; (**B**) degradation profiles of hydrogels made with different concentrations of GelMA in PBS solution with collagenase at 37 °C [47], © 2020 by the authors; (**C**) comparison of the effects of Irgacure 2959 and LAP on the degradation properties of GelMA [48], copyright © 2020 IOP Publishing; (**D**) (i) synthesis of PEG–GelMA composite; (ii) degradation profiles of 5%wt PEGDMA and various ratios of GelMA in DPBS solution with collagenase; (iii) degradation profiles of 20%wt PEGDMA and various ratios of GelMA in DPBS solution with collagenase [49]. Copyright © 2022 Elsevier; (**E**) (i) the degradation behaviors of GelMA-H-nHA hydrogels; (ii) degradation behaviors of P(ACG-GelMA-L)-Mg2^+^ hydrogels [50]. Copyright © 2022 Springer Nature; (* *p* < 0.05, ** *p* < 0.01).

The crosslink density of methacryl groups affects the degradation rate of hydrogels [46]. Increased the prepolymer content also means that more methacryloyl groups are involved in the synthesis of the network, so increasing the ratio of GelMA could slow the degradation rate of the hydrogel. Shie et al. tested the degradation rate of hydrogels synthesized using different contents of gelatin by placing them in a collagenase solution. Hydrogels were made by mixing 0.5% (*w*/*v*) photosensitizer with gelatin at concentrations of 5% (*w*/*v*), 10% (*w*/*v*), and 15% (*w*/*v*), and the residual mass was measured at 2, 4, 12, 24, and 72 h, as well as at 1 and 2 weeks. The images show complete degradation of the 5% gels within one week, near complete degradation of the 10% hydrogels after two weeks, and residual polymer at 9% (*w*/*w*) of the 15% hydrogels after two weeks (Figure 3) [47].

### 4.2. Change of Photosensitizer Type

Photoinitiators are essential reagents for the synthesis of GelMA hydrogels. The type of photocuring agent affects the degradation rate of hydrogels. Xu et al. investigated the effect of photosensitizer type on the physical properties and microstructure of 3D printed pure GelMA hydrogels. It was found that the samples cured with Irgacure 2959 had slightly larger pore sizes, faster degradation, and greater swelling compared to the GelMA samples cured with LAP [48].

### 4.3. Introducing Synthetic Organic Materials

The degradation rate of synthetic materials is lower than that of natural biomaterials. Incorporating synthetic materials can not only enhance the crosslinking strength of GelMA hydrogels but also stabilizes the hydrogel network, which helps slow down the rate of degradation [49]. The reinforced internal structure effectively mitigates the rapid degradation of GelMA, enhances cell adhesion and proliferation, and speeds up the healing process of bone defects.

Kim et al. conjugated GelMA with polyethylene glycol dimethacrylate (PEGDMA) to enhance the mechanical strength of the hydrogel and slow down its degradation rate. The results showed that after 7 days, the composite hydrogel containing 20% (*w*/*v*) PEGDMA retained 20% (*w*/*w*) more mass compared to the composite hydrogel with 5% (*w*/*v*) PEGDMA [50]. Sun et al. created a bilayer bionic artificial periosteum with a GelMA-Hydroxyapatite(nHA) cam layer and a poly(N-acryloyl 2-gobletine) (PACG)-GelMA-Mg layer, enhancing the hydrogel’s robustness through PACG’s hydrogen bonding. The in vitro degradation results showed that the copolymerization of ACG with GelMA prolonged the degradation time of the material to 60 days, which was much longer than the 168 h degradation time of the GelMA hydrogel, allowing the degradation rate of the scaffold to match the rate of bone regeneration. This also demonstrates that hybridizing GelMA with synthetic organic materials is an effective way to reduce the degradation rate of hydrogels [51].

**Table 1:** Summary of degradation abilities of typical GelMA-based hydrogel scaffolds in different solutions.

Type	Material Proportion	Remaining Quantity (*w*/*w*)	Dissolving Solvent
Organic	10% PVA and 10% GelMA [52]	Remaining mass of 62.6, 61.9, and 62.2% with 1, 3, and 5 freeze–thaw cycles	0.01 MPBS solution and 1 μg/mL type II collagenase
Organic	10% GelMA and 1.15% pectin-g-PCL [53]	Remaining mass of 80% on the seventh day	DPBS and DPBS supplemented with 10% fetal bovine serum
Organic	50,000 g/mol PCL and 15%GelMA [54]	A weight loss of 2% by the hybrid scaffold	PBS
Organic	10% GelMA and 1% HAMA [55]	A weight loss of 15% by the hybrid scaffold for 21 days	PBS
Organic	3% GelMA and 2% carboxymethyl chitosan methacryloyl [56]	Almost no degradation in lysozyme and collagenase in 9 days	PBS or PBS supplemented with lysozyme (0.2 mg/mL) or collagenase type I
Organic	10% GelMA and 2% gelatin and chitosan [57]	A weight loss of 25% by the hybrid scaffold for 4 days	PBS with collagenase type Ι
Inorganic	10% GelMA and 5% D-HANR [58]	A weight loss of 12% by the hybrid scaffold for 12 weeks	PBS

## 5. Promotes Bone Mineralisation

GelMA hydrogels are unable to provide many elements, such as calcium, phosphorus, and silicon, needed for bone tissue repair, and their limited osteoconductivity and osteoinduction restrict their application. In situ osteogenic mineralization is a challenge that must be addressed in bone tissue engineering. By combining GelMA with materials that supply essential elements and incorporating substances that improve tissue uptake of these inorganic components, we can preserve the beneficial properties of the raw materials. This approach not only preserves the material’s integrity but also promotes the proliferation and differentiation of osteoblasts, aiding bone formation. Current research focuses on enhancing bone defect repair by incorporating inorganic nanominerals, metals, bone growth-promoting metal ions, growth factors, and exosomes. Several studies, as summarized in Table 2, demonstrate the significant potential of GelMA composite hydrogels in effectively repairing bone defects [29]. The table illustrates the versatility of GelMA composite hydrogels in bone defect repair, with various materials enhancing the matrix substitution. Notably, calcium-based nanoparticles, especially β-tricalcium phosphate and nanoceria, show promising results. Exosomes and cytokines also demonstrate significant potential, with varying degrees of success depending on the specific application. The use of cells, particularly stem cells, appears to be effective, though results vary depending on the cell type and model.

### 5.1. Inorganic Minerals

The inorganic minerals used in the study include hydroxyapatite, nanosilicates, and laponite nanoparticles [75,76]. The introduction of these materials into GelMA hydrogels can promote bone repair and provide nucleation sites for the generation of new bone [77]. However, the introduction of various inorganic mineralized nanomaterials into the system has led to the issue of non-uniform distribution of nanoparticles in the hydrogel, which needs to be solved to effectively support bone regeneration [78].

**Figure 4:**
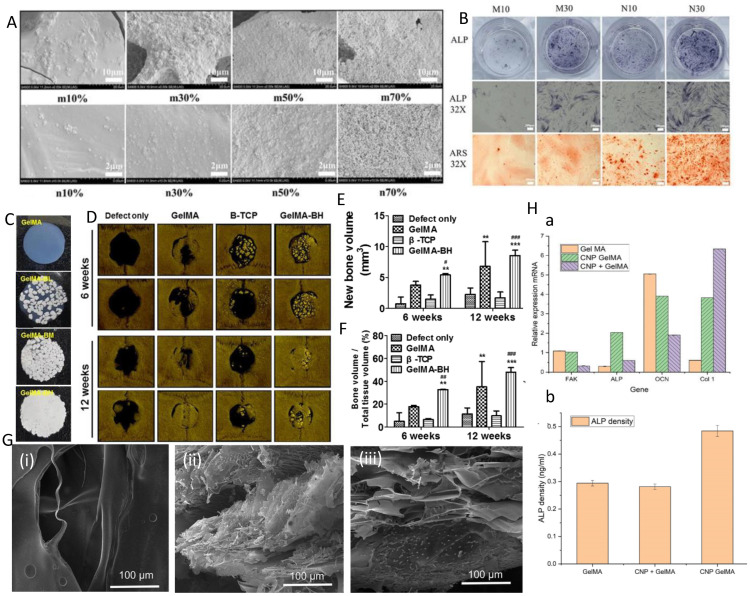
Endowing the hydrogel with osteogenic function with the addition of inorganic mineral materials. (**A**) SEM of GelMA/HAp composite scaffolds; (**B**) ALP staining of GelMA/HAp composite scaffolds [80], copyright © 2022 Elsevier; (**C**) digital images of GelMA-B hydrogels. (**D**) micro-CT analysis after implantation of GelMA-B; (**E**) quantitative analysis of regenerated bone volume with GelMA-B; (**F**) bone volume fraction in total tissue volume with GelMA-B [59]. Copyright © 2019 Elsevier; (**G**) SEM images: (i) GelMA; (ii) CNP (50%) GelMA; (iii) CNP GelMA; (**H**) (a) ALP activity of CNP GelMA; (b) gene expression study of CNP GelMA [81]; (Each group was compared with the control group: ** *p* < 0.01, *** *p* < 0.001; Comparison between GelMA group and GELMA-BH group: ^#^ *p* < 0.05,^##^ *p* < 0.01, ^###^ *p* < 0.001).

Hydroxyapatite (HAp) is a naturally occurring mineral that makes up nearly 50% of the weight of bone, and it contains essential elements, such as calcium and phosphorus. It is widely used as a bone biomaterial [79]. Song et al. mixed lyophilised GelMA powder, with hydroxyapatite modified by a silane coupling agent and fabricated GelMA/HAp composite scaffolds using a DLP 3D printer in the presence of photoinitiator LAP (Figure 4). This approach improves the dispersion of HAp and addresses the bioincompatibility challenge caused by the introduction of small molecules into composites, while closely mimicking the composition of bone. As the results of the study showed, the GelMA/HAp composite scaffolds were able to promote the gene expression of osteogenic proteins, such as ALP, Runx2, BMP2, COL I, and OCN [80].

β-Tricalcium phosphate (β-TCP) is an inorganic material with osteoconductive and osteoinductive properties, which, along with nHA, makes it an ideal material for replacement bone grafts [82]. β-TCP can be dissolved by osteoclasts, and its solubility is similar to that of bone mineral, allowing it to be absorbed by cells and subsequently to repair bones [83]. Lee et al. prepared GelMA-BH, a hydrogel containing β-TCP, and demonstrated that GelMA can exert adhesive forces on β-TCP to ensure that the morphology of the bone is maintained during bone formation. The in vitro cell culture results showed that the expression of bone forming genes, such as COL1, OPN, OCN, and BSP, was higher in the GelMA-BH group than in the control group. The GelMA-BH group showed excellent bone regeneration ability in a rat cranial bone defect model. In the GelMA-BH group, approximately 44.5% of the bone defect area was filled with new bone within 3 weeks, compared to 37.5% new bone formation in the GelMA hydrogel group [59].

Bhattacharyya et al. used a one-pot method to control the distribution of bioactive micro/nanoparticles, based on the nucleation and particle growth of gelatin structures. Amorphous calcium phosphate micro/nanoparticles were prepared using gelatin as a stabilizer prior to the methacrylation reaction. This approach helped to disperse the nanoparticles and solve the problem of inhomogeneous distribution in the system [81]. Expression of mechano-sensing regulated marker is higher than CNP + GelMA hydrogel and similar to pure GelMA hydrogel. Type 1 collagen is significantly higher with all CNP-added hydrogel samples than pure GelMA hydrogel, as observed from Col 1 upregulation. However, OCN gene expression was higher in pure GelMA hydrogel-treated cells (Figure 4H). The ALP density of CNP GelMA group (0.48 ng/mL) is higher than the GelMA group (0.29 ng/mL) and CNP + GelMA (0.28 ng/mL).It shows obvious bone regeneration potential.

Numerous findings also show that by introducing nanoparticles into GelMA hydrogels, osteoblasts can proliferate on the material and extensively mineralize, promoting osteogenic differentiation and, thus, facilitating new bone formation [81,84].

### 5.2. Metal Ions

Metal ions such, as Li^+^, Mg^2+^, and Sr^2+^, can promote bone formation, and these ions are introduced into hydrogels to accelerate defect repair [85,86,87]. However, high concentrations of heavy metal ions can cause rejection reactions in the organism, so trapping and controlling the slow release of these ions are significant challenges.

**Figure 5:**
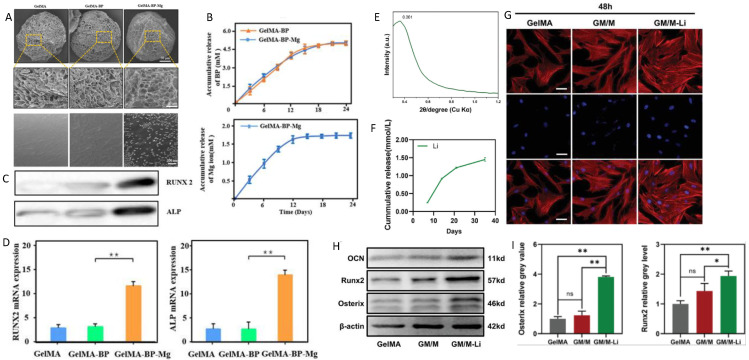
Captures the release of metal ions to promote bone defect repair. (**A**) Scanning electron microscope images of GelMA-BP-Mg microspheres; (**B**) release curves of Mg^2^⁺ and BP from the composite microspheres; (**C**) Western blot of the GelMA-BP-Mg group; (**D**) Q-PCR of GelMA-BP-Mg group [91], copyright © 2021 American Chemistry Society; (**E**) X-ray test of M-Li.; (**F**) Li+ ions released from GM/M-Li hydrogel; (**G**) microfilament skeleton of BMSCs cultured in GM/M-Li hydrogels; (**H**) Western blotting of OCN, Runx2, and osterix proteins; (**I**) quantitative analysis of Runx2 and osterix proteins [92], copyright © 2022 Wiley; (ns ≙ *p* > 0.05, * *p* < 0.05, ** *p* < 0.01).

Magnesium is an important macronutrient in the human body and is involved in metabolism as well as various physiological activities to sustain the body; it is essential for sustaining life. Mg^2+^ activates osteoblast differentiation and promotes angiogenesis after the development of bone defects [88,89]. Zhao et al. designed a bisphosphonate microfluidic hydrogel microsphere that can trap active Mg^2+^ by surface-grafted bisphosphonates (Figure 5). This method avoids the toxic damage caused by the local “high magnesium microenvironment” [90] and the inhibition of the bone regeneration process. As shown in the results, the composite hydrogel promoted the expression of ALP and Runx2 genes and inhibited the activity of osteoclasts, facilitating the repair of bone defects [91].

In many studies, Li^+^ has been shown to favor the proliferation of bone marrow stromal cells (BMSCs) [78,93]. Meanwhile, Li^+^ can accelerate osteogenesis by inhibiting osteoclast differentiation, strengthen BMSC adhesion, and modulate macrophage polarization. Studies have demonstrated that LiCl at a concentration of 5% (*w*/*v*) is effective in inhibiting inflammation [94]. Wu et al. prepared lithium-modified bioglass hydrogels to coordinate bone regeneration in the diabetic microenvironment. Scanning electron microscopy of energy dispersive X-ray spectroscopy revealed that the surface layer of the material possessed a calcium/phosphorus molar ratio similar to that of bone (Ca/P = 1.67), which is favorable for bone mineralization. The results of live–dead cell staining indicated that the GM/M-Li group was more effective in attracting cell adhesion and proliferation compared to the other groups. Additionally, the GM/M-Li hydrogel gradually degraded as the cells proliferated, making it suitable for in situ skeletal reconstruction. The GM/M-Li composite hydrogel upregulated the expression of osteogenesis-related proteins and genes, including OCN, Runx2, and osterix, demonstrating strong osteogenic differentiation [92].

### 5.3. Growth Factors and Cytokines

Cytokines and growth factors are signaling molecules that regulate cellular activities through intercellular communication and play a crucial role in life activities [95]. Osteogenesis-related factors were introduced into the GelMA hydrogel and released at the site of bone defects to promote bone repair.

**Figure 6:**
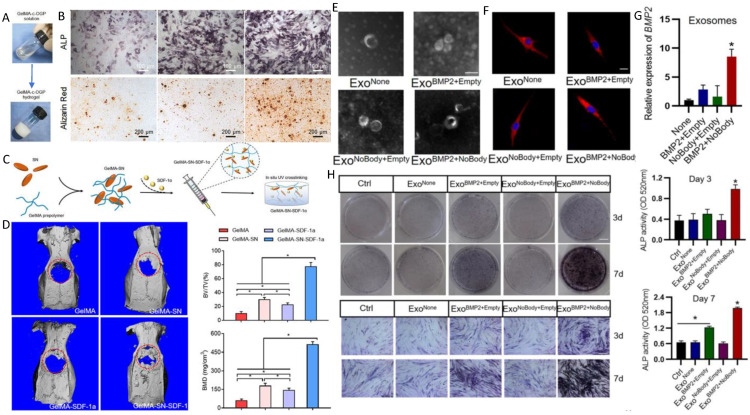
Adding signaling molecules, exosomes, and encapsulated seed cells to repair bone defects. (**A**) Photograph of GelMA-c-OGP solution and GelMA-c-OGP hydrogel; (**B**) the osteogenesis properties of GelMA-c-OGP hydrogel in vitro; (**C**) fabrication of GelMA-SN-SDF-1α hydrogel; (**D**) the area analyzed by micro-CT [72]. Copyright © 2019 Wiley; (**E**) electron microscope image of the engineering exosomes; (**F**) internalization of DiI-labelled EXOs into BMSCs; (**G**) exosome-expressed Bmp2 genes; (**H**) ALP expression and activity were assessed on days 3 and 7 [97]; (* *p* < 0.05).

As an important growth factor, TGF- β1 can regulate the development of bone progenitor cells towards osteogenic differentiation and bone remodeling, and it is involved in bone formation, osteoblast proliferation, and mineralisation by increasing the strength and flexibility of bone [39]. Yamamoto et al. loaded TGF-β1 into GelMA hydrogel in order to achieve slow release of TGF-β1, and subsequently carried out repair of rabbit cranial bone defects (Figure 6). The results of the study showed that the enhancement of bone density in cranial defects was significantly higher when the water content of the hydrogel reached 90 wt% and 95 wt% compared to free TGF-β1 [96].

Osteogenic growth peptide improves the proliferation and differentiation of osteoblasts and accelerates bone formation [98,99]. Qiao et al. co-crosslinked GelMA with osteogenic growth peptide to design a composite hydrogel. Ex vivo experiments verified the osteogenic properties of this composite hydrogel. The results showed that the hybrid hydrogel increased the precipitation of calcium salts in osteoblasts and promoted bone regeneration [72].

Stromal cell-derived factor 1α is a cytokine that has the ability to mobilize hematopoietic stem cells to the bone marrow [100]. Shi et al. loaded stromal cell-derived factor 1α into a laponite–GelMA hydrogel, which demonstrated a potent osteogenic capacity. Micro-CT results showed that after treatment with the composite hydrogel, the newborn bone thickness and density were greater than those of the other groups [101].

In summary, cytokines can be loaded into the hydrogel network to be slowly released at the defect site to promote osteoblast growth, proliferation, differentiation, migration, and healing of bone defects.

### 5.4. Exosomes

Exosomes (Exos) are extracellular vesicles produced by cells that protect internal proteins, mRNAs, microRNAs (miRNAs), and other reactive substances, but which also mediate intercellular information communication by transporting these exogenous substances to receptor cells [102,103]. Studies have demonstrated that gene therapy modalities using mRNA have many advantages over traditional DNA gene therapy in the treatment of bone defects, without the risk of insertional mutagenesis or other genetic damage [104].

Yang et al. co-transfected NoBody and Bone Morphogenetic Protein 2 (BMP2) artificial plasmid into cells to obtain Exos enriched with BMP2 mRNA (Figure 6E). They then loaded these Exos into GelMA hydrogel and evaluated its ability to promote bone regeneration. The Exo-loaded hydrogel was able to highly express BMP2 mRNA and protein. Cell culture results showed that the material was able to promote and maintain the osteogenic differentiation of BMSCs for a long time, and to increase the ALP level and mineralisation level. An animal experiment showed that the material was able to repair the cranial defects well [97]. The conclusion that BMP2 exosome-loaded GelMA hydrogel promotes bone regeneration was also validated by the experiments of Sun et al. [105].

Exosomes (Exos) obtained from mesenchymal stem cells (MSCs) are considered major mediators of the therapeutic efficacy of MSCs and are not subject to the limitations of MSCs therapy. Specifically, Exos derived from BMSCs can recruit BMSCs to damaged areas to promote tissue repair [106]. Yang et al. designed an injectable responsive hydrogel microsphere and demonstrated that the material can promote bone repair through neovascularization by recruiting stem cells. They also noted a negative correlation between GelMA concentration and exosome release rate [107].

### 5.5. Seed Cells

Loading seed cells into tissue scaffolds is a common approach in bone tissue engineering, as these cells can rapidly proliferate and differentiate into osteoblasts, chondrocytes, and other cell types under specific conditions [108]. Some studies have explored 3D encapsulation of cells to evaluate how varying stiffness and porosity affect cell differentiation. They found that cells differentiated towards osteogenesis within a stiffness range of 11–30 kPa, with optimal matrix traction reorganization occurring at 22 kPa. Additionally, MSCs cultured within pores of 100 μm and 150 μm demonstrated the highest osteogenic differentiation capacity [109,110]. Thus, when encapsulating cells, the mechanical strength and internal porosity of the composite hydrogel are crucial considerations.

Mesenchymal stem cells (MSCs) are pluripotent cells with self-renewal, differentiation, and immunomodulatory properties, making them highly potential candidates for repairing bone defects. Shi designed an injectable GelMA–nanohydroxyapatite–nanosilicate composite hydrogel and used it to encapsulate MSCs. The inner pore size of the hydrogel ranged from 150 to 200 μm. The cells were fully spread and proliferated within 7 days, promoting the expression of osteogenic proteins, such as ALP, RUNX2, OCN, and OPN osteogenic proteins [47].

BMSCs are a heterogeneous population of cells derived from mammalian bone marrow and have the ability to form bone in vivo, making them ideal for clinical applications [111,112]. Chai et al. used GelMA hydrogel loaded with both BMSCs and BMP2, and BMP2 was used to stimulate targeted osteogenic differentiation of BMSCs to repair bone defects. The pore size of the hydrogel ranged from 90 to 130 μm, making it suitable for cell growth. Results from the mouse defect model showed that the composite active scaffolds promoted the growth of rat bone trabeculae and accelerated the healing of bone defects [113].

## 6. Other Applications

### 6.1. Endosteal Nerve Repair

Nerve repair is essential for the healing of bone defects. Intrabony nerves not only maintain normal physiological activity by transmitting electrical and chemical signals but also control the further development of bone defects [114,115,116]. Nerve regeneration initiates the repair of bone defects. With the loss of nerve conduction, a number of pathological reactions can occur [117].

**Figure 7:**
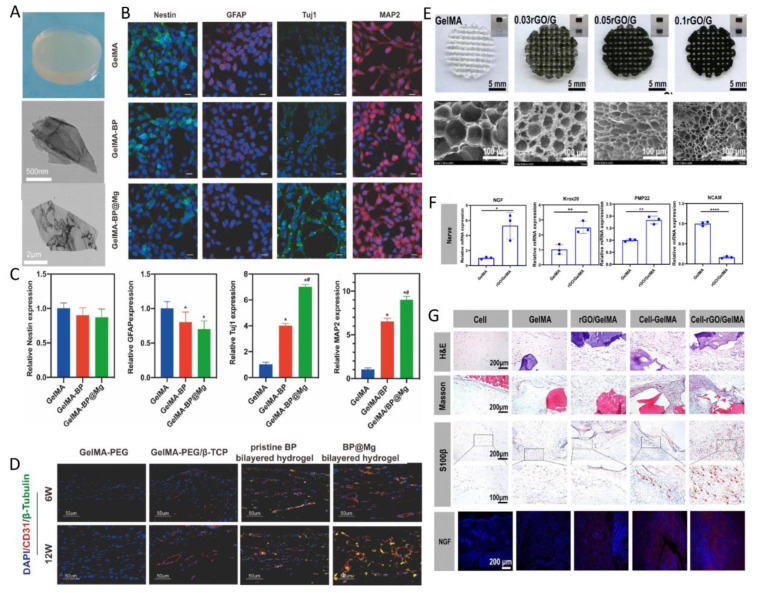
Hybrid BP @ Mg or rGO stimulate nerve repair in bone. (**A**) GelMA-BP@Mg scaffolds; TEM assessment of BP nanosheets; TEM assessment of BP@Mg nanosheets; (**B**) stimulation of neural differentiation in cells on the GelMA, GelMA-BP, and GelMA-BP@Mg hydrogels; (**C**) relative neural-specific gene expression in cells on GelMA-BP@Mg hydrogel scaffolds (*, # Represents marked change in relation to GelMA and GelMA-BP hydrogel, respectively); (**D**) Typical confocal images of CD31 and β3-tubulin co-staining on specimen from the calvarial critical-sized defects [118]; (**E**) macroscopic and scanning electron microscope images of GelMA 3D scaffolds with different rGO concentrations; (**F**) myelination-related assessment of cultured Schwann cells; (**G**) H&E, Masson staining, immunohistochemical staining, and immunofluorescence staining of tissue sections [119]. Copyright © 2013 Royal Society of Chemistry; (* *p* < 0.05, ** *p* < 0.01, **** *p* < 0.0001).

Black phosphorus (BP) nanosheets are 2D nanomaterials composed of phosphorus with biomineralization capacity and electrical conductivity. Hydrogels based on BP nanosheets have been shown to stimulate the differentiation of adipose-derived stem cells (BMSCs) into neuron-like cells, whose degradation contributes to mineralization and accelerates bone reconstruction. Introducing Mg^2^⁺ into BP nanosheets enhances their stability and bioactivity, while BP nanosheets can trap Mg^2^⁺, preventing local overdose. The composite hydrogel was able to upregulate the expression of neural-related proteins in neural stem cells (NSCs), increasing the levels of MAP2 and Tuj-1 (Figure 7). It demonstrates the ability to induce the expression of neuromarkers and enhance the growth of neurites in PC12 cells [118].

Reduced graphene oxide (rGO) is one kind of nanomaterial with a large number of hydrophilic functional groups and a large surface area, obtained by removing some of the oxygen functional groups from reduced graphene oxide (GO), which has high electrical conductivity and enables neuronal cells to migrate and proliferate in response to electrical stimulation [120,121]. Schwann cells (SC) are capable of directing axonal regeneration in proximal segments [122].Co-culturing SCs with BMSCs promotes the expression of nerve growth factor and brain-derived neurotrophic factor (Figure 7E) [123]. Zhang et al. used 3D-printed rGO/GelMA-based hydrogel loaded with SCs and BMSCs for synergistic regeneration of bone and nerves. The results of animal experiments indicated that the material was able to promote the expression of nestin, β-microtubulin III, and MAP2, accelerating the reconstruction of bone defects and nerves within the bone [119].

### 6.2. Intraosseous Angiogenesis

The formation of a functional vascular network is essential for the healing of bone defects. Through the reconstruction of blood vessels in the bone, cells can take up oxygen and nutrients more effectively, thus accelerating the repair of bone defects [124]. The lack of functional vascular network formation has hindered the practical application of bone tissue engineering materials in clinical settings over time [125]. Enabling GelMA composite hydrogel to acquire angiogenic function can accelerate bone reconstruction and promote the healing of bone defects.

**Figure 8:**
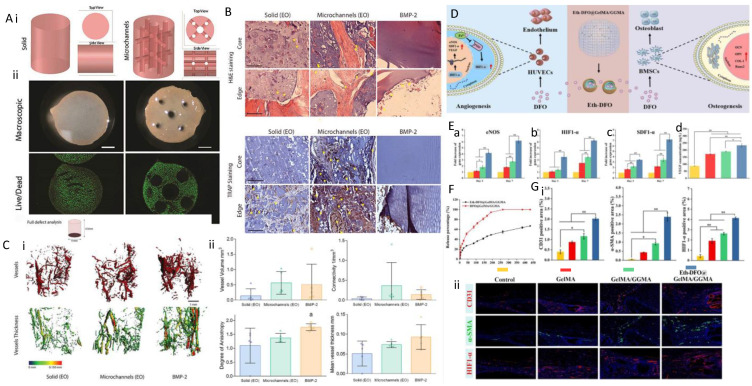
Bionic structure or adding DFO to accelerate bone vascular reconstruction and promote bone defect repair. (**A**) (i) Outline of construct design of microchanneled hydrogels; (ii) microscopic images and live–dead staining; (**B**) H&E and positive TRAP staining after 8 weeks in vivo, revealing areas of red blood cell activity [126]. Copyright © 2018 Elsevier; (**C**) (i) representative 3D reconstructions of vessel formation; (ii) quantification of total vessel volume, degree of vessel anisotropy, vessel connectivity, and mean vessel thickness for all groups; (**D**) mechanistic insight into the Eth-DFO@GelMA/GGMA scaffold-induced osteogenesis and angiogenesis; (**E**) (a) eNOS expression; (b) HIF1-α expression; (c) SDF1-α expression after 3 and 7 days of culture of HUVECs in each group; (d) ELISA assay for VEGF content measurement after 3 days; (**F**) DFO release kinetics from DFO@GelMA/GGMA and Eth-DFO@GelMA/GGMA hydrogels’; (**G**) (i) quantification of the difference in expression of the associated proteins; (ii) immunofluorescence staining of CD31 (red), α-SMA (green), and HIF1-α (red) expression at 8 weeks [127]. Copyright © 2022 Elsevier; (* *p* < 0.05, ** *p* < 0.01).

Daly et al. used 3D printing to create microchannels of pluronic ink encapsulated in BMSCs-loaded GelMA hydrogel to treat critical-sized bone defects by endochondral ossification (Figure 8A). The ability of the scaffolds for angiogenesis and bone defect repair were evaluated using vascular CT analysis and histological staining. The results showed that although their total bone formation level was lower compared to solid cartilage templates, microchannel cartilage templates had a higher level of vascularization within the core region and lower ectopic bone formation [126].

Deferoxamine (DFO) is a small molecule drug that upregulates angiogenesis by triggering the hypoxia-inducible factor (HIF 1-α) pathway both in vitro and in vivo [128,129]. Li et al. designed a bio-scaffold by combining DFO-loaded exosomes with GelMA/gellan gum methacrylate (GGMA) using 3D printing (Figure 8D). The results showed that the composite scaffold achieved sustained release of DFO, activated the HIF1-α signaling pathway, and promoted angiogenesis and bone regeneration [127].

The Wnt/β-catenin signaling pathway is a key regulator of bone homeostasis. Activation of this pathway promotes osteogenic differentiation, while inactivation leads to lipid or chondrogenic differentiation. It has been shown that modifying materials with Wnt3a can increase their bioactivity, improve the efficacy of bone regeneration and angiogenesis, and reduce side effects. Liu et al. incubated fabricated PCL-GelMA scaffolds in Wnt3a for 24 h for cell and animal experiments. The results of animal experiments showed that a large number of new blood vessels formed in the bone tissue sections of the Wnt3a group. Cellular experiments also confirmed its ability to promote the expression of VEGF-A, an angiogenic factor, in ST2 cells, thereby aiding in the reconstruction of bone defects [130].

## 7. Conclusions and Prospects

Conventional fillers used in clinical practice often face limitations. Bone tissue engineering offers new solutions by trying different materials to mimic the structure, composition, and mechanical properties of natural bone for better repair of bone defects [131]. GelMA, a biomaterial of natural origin, is widely used in current research. Due to its unique structure and properties, GelMA can be fabricated into bone repair materials through various processes, such as 3D printing and hydrogel scaffolding [18].

However, GelMA derived from animal sources presents potential immunological concerns. Foreign antigens present in animal-derived GelMA could trigger immune responses, leading to inflammation, biomaterial degradation, or rejection of implanted constructs. To address these issues, advanced purification methods and recombinant gelatin, produced through microbial systems, can be employed to minimize immunogenicity while maintaining the material’s desirable properties.

Researchers need to conduct long-term in vivo experiments to observe the whole process of hydrogel degradation, repair, and reconstruction of bone defects in living organisms.

The development of GelMA-based hydrogels represents a promising frontier in bone tissue engineering, but there are still major challenges from lab to clinical application. The hybridization of GelMA with various components has successfully mitigated some of its inherent limitations, such as low mechanical strength and rapid degradation. Moreover, the introduction of other features, like vascularization and neuralization, further enhances its suitability for bone defect repair, offering new avenues for tissue regeneration [132,133].

Nevertheless, challenges persist. The use of UV light in the crosslinking process raises concerns about potential cytotoxic effects, which could undermine the biocompatibility of the final product. Additionally, the uneven distribution of doped nanomaterials within the hydrogel matrix may lead to inconsistent mechanical properties, failing to match the native tissue’s strength and elasticity. This mismatch could result in stress shielding, where the scaffold bears the mechanical load instead of the surrounding bone tissue, ultimately impeding natural bone regeneration. Furthermore, concerns about the toxicity of photoinitiators, such as Irgacure 2959, persist, particularly in load-bearing applications.

To overcome these obstacles, future research must prioritize optimizing the formulation and processing of GelMA-based hydrogels. This includes developing alternative crosslinking methods that avoid the harmful effects of UV light, ensuring uniform distribution of nanomaterials, and fine-tuning mechanical properties to closely mimic human bone tissue. Although alternatives, like lithium phenyl-2,4,6-trimethylbenzoylphosphinate (LAP), have shown promise, further research is needed to ensure minimal cytotoxicity. Moreover, investigating methods, such as binding osteoinductive growth factors, incorporating bioactive nanomaterials, and utilizing dynamic crosslinking strategies to regulate the release of osteogenic ions, will be crucial for enhancing the osteogenic capacity of these hydrogels, thereby improving their effectiveness in bone regeneration [134].

Regulatory considerations play a critical role in the development and clinical use of gelatin methacryloyl (GelMA) composites for bone tissue engineering. These considerations include material safety and biocompatibility, adherence to manufacturing standards, rigorous preclinical and clinical testing, obtaining market authorization, and ensuring ongoing post-market surveillance and reporting.

As these challenges are addressed, the potential for GelMA hydrogels in clinical applications becomes increasingly promising. With continued innovation and rigorous testing, these materials could soon transition from experimental models to practical solutions for bone defect repair, offering significant benefits in regenerative medicine and improving patient outcomes.

## Figures and Tables

**Table 2:** Summary of GelMA hydrogels and hybrids of different materials to promote bone repair.

Type	Composition of Materials	Model	MA Substitution
Calcium nanoparticles	β-tricalcium [59]	SD rats	81.4 ± 0.4%
Metal ion	Zeolitic imidazolate framework-8 [60]	Wistar rats	60% ± 5%
Cells	Periodontal ligament stem cells [61]	BALB/C-Nude mice	60% ± 5%
Exosome	Human urine-derived stem cell exosomes [62]	SD rats	55–65%
Exosome	Bone marrow mesenchymal stromal cell-derived exosomes [63]	C57BL/6J mice	40%
Cytokine	TGF-β1-CM-KGN [64]	rats	60% ± 5%
Cytokine	BMP-2 [65]	vitro	81.0–93.6%
Cells	MG63 [66]	vitro	72 ± 3.5%.
Calcium nanoparticles	Nanoceria [67]	SD rats	>90%
Nanometal	AuNPs [68]	vitro	53.8 ± 0.5%
Others	Platelet-rich plasma [69]	News Zealand rabbits	81.4 ± 0.4%
Calcium nanoparticles s	Hydroxyapatite doped with cerium ions [70]	vitro	79%
Nanometal	Ti–6Al–4V alloy (TC4) [71]	New Zealand rabbits	19.7 ± 0.7%
Osteogenic peptide	Osteogenic growth peptides [72]	SD rats	70%
Drug	Alendronate [73]	SD rats	81.4 ± 0.4%
Cytokine	Mesoporous bioglass nanoparticles–recombinant human bone morphogenetic protein-2 [74]	SD rats	53.8 ± 0.5%
